# Gene editing in plants: progress and challenges

**DOI:** 10.1093/nsr/nwz005

**Published:** 2019-01-17

**Authors:** Yanfei Mao, Jose Ramon Botella, Yaoguang Liu, Jian-Kang Zhu

**Affiliations:** 1 Shanghai Center for Plant Stress Biology, CAS Center of Excellence in Molecular Plant Sciences, Chinese Academy of Sciences, Shanghai 200032, China; 2 School of Agriculture and Food Sciences, University of Queensland, Brisbane, Queensland 4072, Australia; 3 State Key Laboratory for Conservation and Utilization of Subtropical Agro-Bioresources, College of Life Sciences, South China Agricultural University, Guangzhou 510642, China; 4 Department of Horticulture and Landscape Architecture, Purdue University, West Lafayette, IN 47907, USA

**Keywords:** CRISPR, Cas9, genome editing, base editing, gene targeting, crop breeding

## Abstract

The clustered regularly interspaced short palindromic repeat (CRISPR)-associated protein 9 (Cas9) genome editing system is a powerful tool for targeted gene modifications in a wide range of species, including plants. Over the last few years, this system has revolutionized the way scientists perform genetic studies and crop breeding, due to its simplicity, flexibility, consistency and high efficiency. Considerable progress has been made in optimizing CRISPR/Cas9 systems in plants, particularly for targeted gene mutagenesis. However, there are still a number of important challenges ahead, including methods for the efficient delivery of CRISPR and other editing tools to most plants, and more effective strategies for sequence knock-ins and replacements. We provide our viewpoint on the goals, potential concerns and future challenges for the development and application of plant genome editing tools.

## INTRODUCTION

Genetic diversity is a key resource for genetic research and trait improvement in plants. For thousands of years, plant domestication relied on natural variations to select for favorable genetic changes. During this process, the generation of genetic variants was completely uncontrollable and largely depended on the environment of plant cultivation.

In order to create new varieties, breeders have used different methods to introduce heritable mutations into plant genomes. In the past century, the use of various mutagens enabled rapid generation of large pools of genetic variation. Chemical compounds and irradiation are common mutagens used in traditional breeding programs to induce random mutations. However, these methods have several drawbacks, including the non-specific nature of the generated mutations, the large amount of nucleotides simultaneously mutated and sometimes the deletion, duplication or rearrangement of large genomic fragments [1]. As a consequence, the identification of mutations of interest is a long and labor-intensive process. In addition, random mutagenesis methods are usually less effective for trait improvements in polyploid crops, given their formidable genetic redundancy.

The development of sequence-specific engineered endonucleases, the mega-nucleases, zinc finger nucleases (ZFNs), transcription activator-like effector nucleases (TALENs) and type II clustered regularly interspaced short palindromic repeat (CRISPR)/CRISPR-associated protein 9 (Cas9), has paved the way for targeted gene editing in plant genomes [2]. These programmable nucleases enable the generation of double-stranded DNA breaks (DSBs) in a site-specific manner. In eukaryotic cells, the induced DSBs can be repaired either via the error-prone end-joining pathway or via the error-free homology-directed repair (HdR) pathway [3]. Both pathways can be harnessed to introduce gene modifications at the target loci. However, the choice of repair pathway depends on many factors including the phase of the cell cycle, the nature of the DSB ends and the availability of repair templates [4].

The most powerful gene editing tool available, the well-developed CRISPR/Cas9 system, is an RNA-directed DNA endonuclease adapted from the bacterial immune system [5]. It is composed of a CRISPR RNA (crRNA) molecule for target recognition, a trans-activating crRNA (tracrRNA) for crRNA maturation and a Cas9 protein for DNA cleavage. The crRNA-tracrRNA duplex has been artificially fused into a chimeric single guide RNA (sgRNA) to direct DNA cleavage by the Cas9 protein [6]. Given the simplicity of this two-component CRISPR/Cas9 system, it has been widely adopted for research in eukaryotic organisms, including plants [7,8]. Theoretically, any DNA molecule with sequence complementarity to the first 20 nt of the sgRNA can be a target, but DNA cleavage is only permitted when a G-rich (NGG) protospacer adjacent motif (PAM) is identified at the 3′ end of the DNA targets (protospacer) [9]. The original CRIPSR system was developed from the bacterium *Streptococcus pyogenes* (SpCas9), but there are now many Cas9 orthologs identified from different bacterial genomes with diverse properties [10]. For example, a smaller Cas9 derived from *Staphylococcus aureus* (SaCas9) as well as a Cas9 derived from *Streptococcus thermophilus* (StCas9) were shown to work efficiently in plants [11]. In addition to type II CRISPR/Cas9 systems, type V CRISPR/Cas12a (also known as Cpf1) systems have also been harnessed for plant gene editing. Type V Cas12a systems are quite different from Cas9 systems in three aspects. First, they recognize T-rich PAM sequences (TTTN or TTN), which are located just upstream of the non-complementary strand of the target. Second, Cas12a proteins produce DSBs with 5 nt 5′ overhangs instead of the blunt ends produced by type II CRISPR/Cas9 systems. Third, Cas12a can process its own crRNAs from primary transcripts of CRISPR arrays and no tracrRNAs are required for crRNA maturation [12,13]. The unique properties of CRISPR/Cas12a systems make them a good complement to CRISPR/Cas9 systems. Moreover, to further expand the gene editing toolkit, engineered Cas9 variants with altered PAM sequences and improved cleavage specificity have been developed [14]. For example, phage-assisted continuous evolution has been used to produce an ‘evolved’ xCas9 protein that can recognize a broad range of PAM sequences and reduce the generation of off-targets in the human genome. Recently, a more efficient SpCas9 variant compatible with ‘NG’ PAM was obtained via structure-directed evolution strategies [15], and this Cas9-NG-derived editing tool has been shown to be effective in plants [16]. Many studies have shown that new tools originally developed for animal systems can also work efficiently in plant cells [17–19].

The outcomes of CRISPR-induced DSBs repaired by the end-joining system are mostly small insertions and deletions (indels), in the absence of donor templates [20]. Hence, CRISPR tools are most commonly used as a biological mutagen to induce the generation of out-of-frame mutations in genes of interest. This application of CRISPR/Cas9 largely facilitates the production of heritable gene mutations for reverse genetics studies and crop breeding, especially when multiple genes need to be mutated simultaneously. Although targeted gene replacements or integrations can also be obtained using CRISPR/Cas9 systems, the frequency is still very low.

Taking advantage of the high mutagenesis efficiency of CRISPR/Cas systems, another important application of this technology is in the realm of forward genetics studies. The development of CRISPR/Cas systems allows the simultaneous and random modification of functionally redundant or related genes by customizing a guide RNA library. So far, CRISPR/Cas systems have been harnessed to generate customized mutation libraries at a genome scale in human cell lines and many other species including rice [21–23]. The size and nature of the customized guide RNA library can be very flexible, ranging from a few genes in a specific gene family to a large number of genes suspected to be involved in broad genetic pathways, depending on the research purpose. Compared to the traditional random mutagenesis methods, the use of CRISPR mutation libraries can be very focused, hence decreasing work load and cost in genetic screens. Moreover, unlike random mutagens, CRISPR systems recognize their targets via base pairing, therefore gene mutations that induce interesting phenotypes can be readily traced by identifying the corresponding guide RNA sequences.

Despite the enormous potential of CRISPR and other gene editing tools, significant challenges remain if this potential is to be fully realized. Some of the challenges have to do with technical innovations needed for effective delivery of the editing tools and for precise gene editing, while others come from the social environment, such as government policies and public acceptance.

## CONCERNS ABOUT GENE EDITING

### Are gene-edited plants ‘genetically modified organisms’?

Since the first use of CRISPR/Cas9 for plant gene editing in 2013, this powerful tool has been quickly adopted by the research community and greatly boosted the study of plant genetics [24]. In contrast, its application in crop breeding is still hampered by several concerns. There is controversy surrounding the technology in parts of the world, not the least of which focuses on whether gene-edited plants should be considered ‘genetically modified organisms’ (abbreviated commonly as GMOs), as defined previously for transgenic organisms.

All trait-improved crops have arisen from genetic and/or epigenetic variations. To accelerate the generation of variants for human consumption, physical or chemical mutagens, such as radiation and ethyl methanesulfonate, have been widely used to induce changes in plant genomes for many years [25]. In 1983, the first transgenic plants were generated using disarmed *Agrobacterium tumefaciens* strains, whose tumor-inducing elements in Ti plasmids had been replaced by antibiotic resistance markers [26]. This technology enabled the integration of exogenous DNA fragments, called transgenes, into host plants to confer a new property, e.g. herbicide or insect resistance, yield increase or quality improvement. Organisms produced in this way are called GMOs. Although these transgenic crops have contributed to the improvement of agriculture production, they became associated with unsubstantiated concerns over food and environmental safety. As a consequence, strict regulatory frameworks and exhaustive risk assessment processes were imposed on GMOs in many countries [27]. With the emergence of gene editing tools, there is a need to reconsider the current definition of GMOs and corresponding regulatory frameworks, since the genome modifications achieved by gene editing methods are very different from those of transgenic technology. Firstly, most of the CRISPR-induced gene mutations are small indels rather than large fragment insertions or rearrangements [20]. Such small indel variations are frequently present in plants grown under natural conditions and can also be induced at a large scale using radiation or chemical mutagens [25]. Second, unlike traditional GMO plants, which require the presence and stable inheritance of transgenes in the genome, trait-improved plants created using CRISPR and other gene editing tools can be transgene-free. To obtain transgene-free plants, CRISPR constructs can be transiently expressed in plant cells, without any DNA integration into the genome, or CRISPR constructs can be stably incorporated and expressed but then removed by genetic segregation. Alternatively, CRISPR systems can be delivered into plant regenerative cells without DNA constructs using *in vitro* transcripts or ribonucleoproteins (RNPs).

There is no internationally accepted regulatory framework for gene editing. As an example of two opposite regulatory policies, the US Department of Agriculture has determined that gene-edited crops are exempt from regulation [28], while the European Court of Justice has recently ruled that gene-edited products should be treated like traditional GMOs, which are subjected to very strict regulation in the European Union [29]. For many countries, a clear regulatory policy has not been developed for gene-edited crops. Gene-edited plants can be considered as products of biological mutagenesis, much like chemical and radiation mutagenesis widely used in conventional plant breeding. In our opinion, transgene-free gene-edited plants should be treated in the same way as plants bred by conventional chemical or radiation mutagenesis, and should not be subjected to special regulatory policies. An important consideration in the implementation of regulatory policies is the ability to identify the regulated organisms (GMOs). The widespread adoption of genome editing technologies bring serious technical challenges to the regulatory authorities, as it can be impossible to differentiate the edited events from natural or chemical/radiation-induced mutants.

Much like the nuclear fission technology that can be used to generate electricity to benefit mankind or to make nuclear bombs for human destruction, the revolutionary CRISPR gene editing technology can bring enormous benefits to humans through applications in crop breeding, or cause fear and ethical disaster when used to make ‘CRISPR’d babies’ [30]. Clearly, science-based regulatory policies that treat gene-edited crops the same way as crops from conventional mutagenesis breeding are needed to encourage the application of gene editing technologies for crop breeding to feed the growing population in the world.

### Off-target mutations

The specificity of CRISPR/Cas9 systems is a major concern in the application of CRISPR tools for targeted gene editing, especially in the field of gene therapy in humans. Some studies in mammalian cells and other systems have shown that Cas9/sgRNA complexes often have the ability to cut DNA sequences with an imperfect match to the guide sequences [31,32], while others have shown moderate or low off-target activity [32,33]. In plants, when whole-genome sequencing was applied to detect off-target mutations in *Arabidopsis* [34], rice [35,36] and tomato [37], very limited off-target effects were identified. Any potential off-target sites can be largely avoided by designing guide RNAs with high specificity using software tools, such as CRISPR-P [38] and CRISPR-GE [39]. In general, the nucleotides in the PAM and PAM-proximal sgRNA sequences are crucial for target recognition, while PAM-distal sequences can tolerate some, but not many, mismatches [32]. Moreover, the specificity of CRISPR systems can be further improved by using engineered Cas9 variants, such as enhanced-specificity Cas9s (eSpCas9) [40], high-fidelity Cas9s (Cas9-HF) [41] and xCas9 [42].

Compared to medical applications, the off-target activity of CRISPR systems in plant cells is less concerning. In gene function studies in plants, off-target effects might interfere with the analysis and interpretation of the results, making it necessary to ascertain whether there is any off-target mutation in the genome affecting the phenotype of interest. Any unwanted mutations can be segregated out through genetic crosses. In a segregating population, a correlation between the phenotype and genotypes of on-target and off-target can be established in progenies. When gene editing tools are applied to crop breeding, off-target mutations may have either a negative, no (neutral) or positive effect on agronomic traits. Plants with negative-effect mutations are naturally discarded during the breeding/selection process or, alternatively, the negative-effect mutations can be segregated out during sexual reproduction. However, if the off-target mutations have neutral or positive effects on the trait(s), they can be retained in the newly bred lines. Therefore, similar to traditional breeding using physical and chemical mutagens where numerous mutations are generated, there is no need to be concerned with any off-target effects, because the breeding process selects for plants with mutations having positive or neutral effects, regardless of whether the mutations are due to on-targets or off-targets.

## CHALLENGES AND PROGRESS IN PLANT GENE EDITING

Engineered CRISPR systems are quickly becoming more efficient, flexible and precise to meet multiple requirements for targeted gene modifications. Although these developments are paving the way to design dream plants for the future, many challenges still remain.

### How to deliver gene editing tools into regenerative plant cells?

Since the first application of a disarmed *Agrobacterium* strain for plant transformation in 1983, the *Agrobacterium*-mediated genetic transformation method has become the most commonly used gene delivery technique in a number of plant species. This technique is very robust and simple to use, especially for *Arabidopsis* and some related crucifers. Transgenic plants can be obtained efficiently via sexual propagation by screening for antibiotic-resistant seedlings from *Agrobacterium*-infected flowers. For plant expression, the engineered CRISPR systems need to be transcribed using plant-optimized gene transcription modules and inserted into the T-DNA region of a binary vector [43]. Although the introduced CRISPR constructs are very effective, it is almost impossible to avoid the generation of chimeras in the T1 generation, unless successful genome modifications are produced at the zygote stage [44,45]. Therefore, to generate heritable genome modifications, the CRISPR cassettes need to be expressed in germline cells or meristematic cells that have the potential to generate germline cells [46]. In general, promoters with strong expression in meristematic cells are preferred to generate heritable gene mutations in the progeny of first-generation transgenic plants. It also worth mentioning that the *in plant*a expression of the introduced CRISPR/Cas systems can be regulated not only that the transcriptional level, but also at the post-transcriptional level [47]. Therefore, the chance of obtaining heritable gene mutations can be further improved if the post-transcription gene silencing pathway is suppressed.

However, for most other plants, the flower dipping *in planta* transformation method is not feasible. Therefore, it is necessary to regenerate transgenic plants from explant-derived calli. *Agrobacteri*a and biolistic particle bombardment both can be very effective in delivering CRISPR constructs into cultured plant cells [48]. Considering that genetically modified plants are regenerated from transformed cultured cells, the gene editing efficiency of CRISPR systems can be optimized either by using strong constitutive promoters or by extending the culture period [49]. Transgenic plants containing homozygous or biallelic gene modifications can even be obtained in the first generation after tissue culture, accelerating the application for crop breeding [35,50].

When these plant genetic transformation methods are used to deliver the CRISPR/Cas9 constructs into plant cells, some people may be concerned about the presence of transgenes during the editing process, even though the final products can be made transgene-free [51]. To avoid such concern, an alternative method is to deliver *in vitro* transcripts of CRISPR modules or assembled Cas9 RNPs into regenerative cells [52]. However, due to the protective effect of the cell wall, direct cell injection or transfection methods, such as microinjection, lipofection or electroporation, are not usually suitable for intact plant cells. Nevertheless, there are solutions to this problem, as in the case of lettuce, where Cas9 RNPs can be introduced into wall-less protoplasts followed by tissue regeneration [53]. Direct delivery of Cas9 RNPs or In vitro Transcription (IVTs) into young embryos of maize and bread wheat has also been achieved using biolistic bombardment [54,55]. Although the use of IVTs and RNPs alleviates concerns about the random integration of foreign DNA fragments, the procedure of tissue regeneration from protoplasts and the identification of gene-edited plants from bombarded embryos can be very costly and laborious. So far, these transgene-independent techniques are only feasible with very few plant species and varieties.

### How to deliver gene editing tools into non-transformable plants?

The biggest hurdle for the application of plant gene editing technologies is the lack of a powerful cell delivery method into reproductive cells, which could be readily and widely applied to diverse plant species, especially recalcitrant species that are difficult or impossible to regenerate through tissue culture.

In the past decade, nanotechnology has had a profound impact in a variety of fields, including manufacturing, energy and medicine. However, its application to plant science, especially using nanocarriers to deliver chemicals and biomolecules into cells, is still a new research area. Compared to mammalian cell systems, nanoparticle (NP)-mediated plant biomolecule delivery is more challenging, owing to the presence of the plant cell wall. Several NPs, such as carbon nanotubes [56], mesoporous silica NPs [57] and metal/metal oxide NPs [58] can traverse the plant cell wall and be taken up directly by plant cells, while other NPs, such as gold NPs, magnetic NPs and some composite NPs, require external aids to assist in their penetration [59]. NP uptake and permeability throughout plant tissues is limited by cell wall pore diameters. The cell wall is commonly thought to exclude particles >5–20 nm; however, NPs ≤ 50–200 nm were reported as cell wall permeable with external aids [60].

To utilize NPs for *in planta* genetic engineering, the core problem is how to deliver preloaded NPs into plant reproductive cells. Recently, magnetofection, a method using magnetic NPs as DNA carriers, has been used to deliver foreign DNA into cotton pollens [61]. After pollination using the transfected pollens, stably transformed plants were obtained in progenies at a frequency of about 1%. If reproducible, this study might open the door to generate heritable gene modifications in a wide range of flowering plants using magnetofected pollens. Nevertheless, a possible limitation of this method is its reliance on the presence of multiple pores in the pollen for the NPs to enter. In addition to pollen grains, other germline cell-containing tissues and meristematic tissues may also be targeted for NP-mediated delivery of genes or proteins, since gene modifications in these tissues may be transmitted to the progeny, although efficient delivery of NPs into these reproductive cells can be very challenging, considering that they are usually hidden by multiple outer tissue layers.

### How to do multiplex editing with high efficiency?

With the explosion of genome sequencing data, gene functional studies have become more and more dependent on reverse genetic strategies. In plants like *Arabidopsis*, forward genetics approaches have led to the identification of the genetic functions of many genes over recent decades [62]. However, phenotypes contributed by redundant genes are usually missed by forward genetics screens. The use of CRISPR has allowed the simultaneous targeting of multiple genes, facilitating the analysis of functionally redundant genes by reverse genetics.

**Figure 1.**
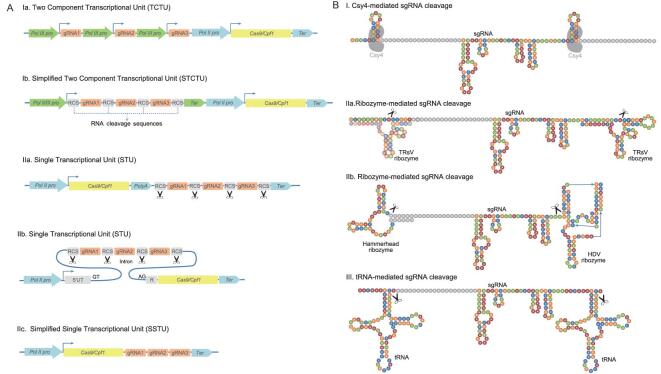
Multiplex gene editing systems in plants. (A) Vector systems developed for multiplex gene editing in plants. Pol II/III pro: RNA Pol II- or III-dependent gene promoters; gRNA: guide RNA; Cas9/Cas12a: Cas9 or Cas12a coding sequences; Ter: terminator; RCS: RNA cleavage sequences; Poly A: polyadenylation sequences; 5′UT-R: 5′ untranslated region. (B) Strategies used for co-expressing multiple guide RNAs within a single RNA transcript. Csy4: CRISPR/Cas Subtype Ypest protein 4; TRsV ribozyme: a ribozyme derived from the tobacco ringspot virus; HDV ribozyme: a ribozyme derived from Hepatitis delta virus; tRNA: transfer RNA.

CRISPR systems can be engineered to target multiple genes with homologous sequences using only one or two sgRNAs for target recognition. However, if no sequence similarity exists in the target genes, specific sgRNAs will be required for each target. In animal systems, the coding sequences or transcripts for multiple sgRNAs can be separately prepared and mixed together before cell delivery [63]. However, the common plant transformation methods, such as *Agrobacterium*-mediated genetic transformation and biolistic bombardment, are seldom used for co-transformation of multiple vectors due to the low efficiency of co-delivery. To achieve multiplex targeting, it is necessary to co-express multiple sgRNAs within a single construct. The two most common strategies (Fig. 1) to achieve this purpose are explained below.

#### Two-component transcriptional unit systems

In this strategy, the expression of Cas9 and sgRNAs is driven by separate transcriptional regulatory units. To achieve maximal expression in plants, strong promoters are usually recommended for the sgRNAs and Cas9. RNA polymerase (Pol) II-dependent promoters with strong expression in reproductive cells or corresponding ancestor cells are good candidates for the control of Cas9 expression. In contrast, the non-coding sgRNAs are more suited for transcriptional control by Pol III-dependent promoters, such as the U6 and U3 promoters, which produce very precise transcripts and are highly active in the majority of cell types [8]. Many research groups have shown the potency of CRISPR-directed multiplex targeting by stacking a set of sgRNA expression modules into a single binary vector [50,64]. Successful assembly of up to eight sgRNAs within single CRISPR vectors has been reported [50,65,66].

To enable the efficient co-expression of multiple sgRNAs, they can be expressed separately using different Pol III-dependent promoters [50,67]. Considering that the multiple sgRNA modules are usually connected in tandem in plant expression vectors, such a design helps to reduce the sequence repetitiveness of CRISPR constructs and thus reduce the potential for silencing. Another option is to assemble the multiple sgRNAs into a single transcription unit. In this case, the primary transcript must be processed to generate multiple mature sgRNAs [65,66]. This strategy makes use of self-cleaving RNAs or cleavable RNA molecules, such as the csy4, ribozyme and tRNA sequences, to process the primary transcript into multiple sgRNAs (Fig. 1). Either of the two strategies described above are quite efficient in plants, highlighting the robustness of CRISPR systems to achieve multiplex gene editing.

#### Single transcriptional unit systems

To express functional CRISPR/Cas9 systems in specific plant cells or developmental stages, it is important to synchronize the expression patterns of sgRNA and Cas9, especially when targeting multiple genes. This can be accomplished by using inducible, or tissue- and development-specific promoters to drive the expression of sgRNA and Cas9 within a single primary transcript. To allow processing of the primary transcript into functional sgRNA and Cas9 subunits, the above-mentioned self-cleaving RNA molecules can be constructed into the introns or untranslated regions to direct the generation of functional CRISPR components [68,69]. However, several studies have reported successful targeting of multiple genes in rice using an even simpler single-transcriptional unit (STU) system, without any cleavable RNA sequences [70,71]. The editing efficiencies of the simplified STU systems were comparable to those separately expressing sgRNA and Cas9 transcripts. Although the mechanism behind this observation is still not very clear, it is possible that sgRNAs can be released from the primary transcripts with the help of Cas9 and/or plant endogenous RNA processing proteins. The use of these STU systems is expected to further increase the flexibility and throughput of CRISPR systems for multiplex gene editing.

### How to perform precise gene editing in plants?

Targeted DNA sequence integration or replacement, also known as gene targeting, is a precise gene editing technology based on HdR. The HdR process facilitates the exchange of homologous DNA fragments between parental chromosomes to increase genetic variation. To harness this process for precise gene modification, the most popular strategy is to introduce DNA templates flanked by sequences homologous to the target site into reproductive cells. In recent decades, gene targeting has been widely adopted for genetic engineering in mammalian cells; however, due to the low HdR frequency and the lack of an efficient donor DNA delivery method, it has rarely worked in plants [72].

**Figure 2.**
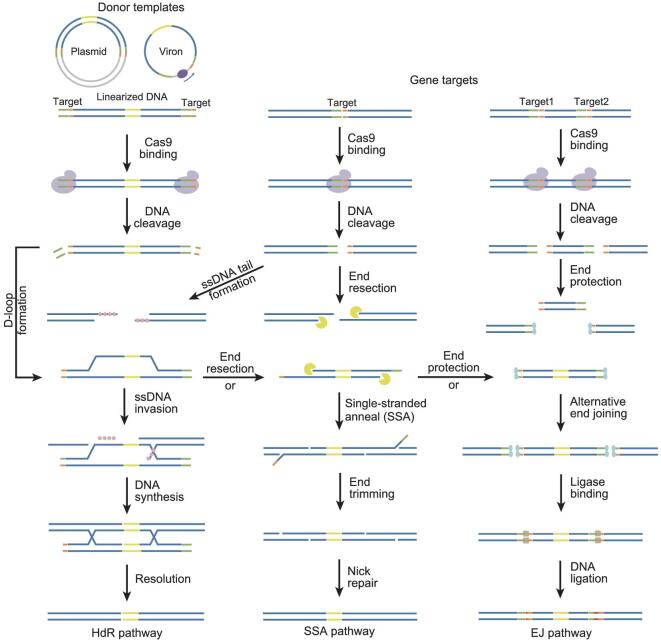
Precise gene editing using CRISPR systems. In the presence of a donor DNA template, precise gene editing can be accomplished via three different DSB repair pathways. The donor templates are supplied mainly in three forms: linearized double-stranded DNA, circular plasmids and single-stranded DNA (ssDNA) replicons. To facilitate the release of donor templates from the backbone, an sgRNA target is usually fused to each end of the DNA template. When the CRISPR-mediated cleavage of the gene target and donor template are synchronized, targeted gene replacement can happen via three different repair pathways. For HdR and Single-stranded Anneal (SSA) pathways, the integration of donor templates into gene targets can be seamless owing to the base pairing between their homologous sequences. With the end-joining (EJ) pathway, indels are usually induced at the junctions of swapped sequences. The sgRNA binding sites within the gene targets and donor templates are indicated in green. The PAM motifs are shown in orange. The anticipated gene mutations within the donor templates are highlighted in yellow. The indels induced by end-joining repair are shown in red.

A critical step to initiate the HdR repair process in plant cells is to induce the generation of a DSB at the targeted gene locus [73]. With the development of CRISPR, this step can be achieved easily in a broad variety of cell types. CRISPR-mediated precise gene editing has been achieved successfully in multiple plant species (Table 1). A commonly used strategy is to engineer a donor template with the desired gene sequence change(s) between two homology arms [74]. To avoid the cleavage activity of CRISPR systems on the donor template, the targeted site within the donor template is usually mutated. The length of the homology arms flanking the inserted or replaced fragments need to be optimized in the context of the targeted genes. The donor template can be included in the plasmid containing the CRISPR cassette or built into a separate plasmid for delivery depending on the transformation method. True gene targeting events are usually screened in transgenic lines using selection marker genes or PCR-based genotyping approaches (Fig. 2).

**Table 1.** Summary of CRISPR-based plant precise gene editing systems.

Species	Transformation	Donor	Promoter	Nuclease	Target	Selection	GT efficiency	Reference
*Arabidopsis*	*Agrobaterium*	BeYDV replicons	PcUBI	Cas9/Cas9n	GL1	None	0.0%	[105]
		T-DNA		Cas9			0.1%	
*Arabidopsis*	Biolistic	CaLCUV replicons	35S	ZFN	ADH1	None	4.3%	[79]
*Arabidopsis*	*Agrobaterium*	T-DNA	PcUBI	Cas9/Cas9	ADH1	Allyl alcohol	0.1%	[75]
*Arabidopsis*	*Agrobaterium*	T-DNA	PcUBI	Cas9/Cas9n	ALS	Imazapyr	0.14–0.3%	[82]
			AtYao				0.1%	
			AtEC1.1/1.2				0.05–0.97%	
*Arabidopsis*	*Agrobaterium*	T-DNA	AtDD45	Cas9	ROS1	None	6.3–8.3%	[81]
					DME		5.3–9.1%	
Tobacco	*Agrobaterium*	BeYDV replicons	35S	TALEN/Cas9	ALS	Kanamycin		[79]
				ZFN	GUS		NA	
Tomato	*Agrobaterium*	BeYDV replicons	35S	Cas9	ANT1	Kanamycin	3.65–11.66%	[106]
				TALEN			4.67–9.65%	
		T-DNA		TALEN			1.3%	
Tomato	*Agrobaterium*	BeYDV replicons	SlUBI10	Cas9	ctisto	None	25.0%	[107]
Soybean	Biolistic	DNA vector	EFLA2	Cas9	DD20	Hygromycin	4.6%	[108]
					DD45	Hygromycin	3.8%	
Potato	*Agrobaterium*	BeYDV replicons	35S	Cas9	ALS	Kanamycin	32.2%	[109]
				TALEN			34.5%	
Maize	Biolistic	DNA vector	ZmUBI	Cas9	ALS2	Chlorsulfuron	0.2–0.4%	[76]
		ssDNA						
	*Agrobaterium*	T-DNA			LIG1	Bialaphos	2.5–4%	
					ALS	Chlorsulfuron	4.2%	
Rice	Biolistic	DNA vector	ZmUBI	Cas9	ALS	Bispyribac	90.6%	[77]
	*Agrobaterium*						75.0%	
Rice	*Agrobaterium*	T-DNA	35s	Cas9	ALS	Bispyribac	0.147–1%	[110]
Rice	*Agrobaterium*	WDV replicons	ZmUBI	Cas9	GST		19.4%	[42]
					ACT1		7.7%	
		T-DNA			GST		6.8%	
					ACT1		0.0%	
Rice	*Agrobaterium*	Chimeric sgRNA	OsUBI	Cas9	ALS	Bispyribac	2.1%	[111]
Rice	Biolistic	DNA vector	35S	FnCas12a	CAO	Hygromycin	3–8%	[112]
				LbCas12a			0–3%	
Rice	Biolistic	DNA vector	ZmUBI	Cas9	NRT1.1B	None	6.7%	[78]
Rice	Biolistic	DNA vector	ZmUBI	LbCas12a	ALS	Bispyribac	11.1%	[113]
Maize	Biolistic	DNA vector	ZmUBI	Cas9	ARGOS8	None	0.9%	[114]
Wheat	Biolistic	WDV replicons	ZmUBI	Cas9	MLO	GFP	3.2–6.4%	[115]
					EPSPS		4.7%	

BeYDV: bean yellow dwarf virus; CaLCUV: cabbage leaf curl virus; WDV: wheat dwarf virus; ssDNA: single-stranded DNA; PcUBI: parsley ubiquitin promoter; SlUBI10: tomato ubiquitin10 promoter; EFLA2: soybean elongation factor gene promoter; ZmUBI: maize ubiquitin promoter.

The first described CRISPR-directed gene targeting event was achieved in the *Arabidopsis Alcohol Dehydrogenase* (*ADH1*) gene via *Agrobacterium*-mediated genetic transformation [75]. To facilitate the recombination process, additional sgRNA target sequences were added to both ends of the donor template, located within the same vector as the CRISPR cassette. In total, two stable gene-targeted (GT) lines were identified out of approximately 1400 T2 seedlings. A similar editing frequency (0.2–0.4%) was obtained when the maize *Acetolactate Synthase* (*ALS*) gene was targeted using three different donor templates (a double-stranded vector and two single-stranded oligonucleotides) [76]. Each template was introduced into immature embryos using biolistic bombardment along with the CRISPR construct at a 1:1 ratio. However, *Agrobacterium*-mediated transformation achieved much higher gene targeting frequencies (2.5–4%) of the endogenous *LIGULELESS1* (*LIG1*) locus. Precise GT was achieved at high efficiency in the rice *ALS* gene (48 homozygous lines were obtained from 52 herbicide-resistant calli) when two sgRNAs were used for target recognition. Further, the ratio of CRISPR construct and donor template DNA was adjusted to 1:20 for plant transformation via biolistic methods [77]. This strategy has been used to modify other plant endogenous genes, although at a much lower frequency. The gene targeting frequency of the rice nitrate transporter gene NRT1.1B reached 6.7% in T0 plants, even though no selection marker was used [78].

Unlike particle bombardment strategies, which are quite flexible at optimizing the ratio of CRISPR components and donor templates, the use of *Agrobacterium*-mediated transformation for plant gene targeting is limited by the abundance of DNA molecules delivered into plant cells. To overcome this problem, Baltes *et al.* first reported the use of geminiviral replicons for plant gene targeting [79]. The engineered DNA replicons contain two cis-acting elements, a long intergenic region and a short intergenic region, which are recognized and regulated by the replication-initiation proteins Rep and RepA. Once delivered into plant cells using *Agrobacterium*-mediated transformation, the engineered replicons could produce up to ∼6000 copies of the gene per cell within 5 days through rolling-cycle replication [79]. Due to the size limit of the DNA cargo, this viral replicon was used for the delivery of sgRNAs and donor templates (<800 bp), but not the Cas9 gene, into host plant cells. GT plants have been regenerated from viral replicon-infected rice, wheat and some Solanaceous cells [42,80]. Nevertheless, the identification of the GT plants still relied on selection for antibiotic resistance genes at the targeted loci and the few gene targeting events that did not rely on selection markers displayed extremely low frequencies, limiting the usefulness of these approaches.

However, in *Arabidopsis*, two studies have shown that CRISPR systems using Cas9 under the control of the egg cell- and early embryo-specific DD45 gene promoter can improve the frequency of targeted gene knock-in and sequence replacement via HdR [81,82]. In one of the studies, the donor templates and the CRISPR cassettes were constructed into the same vector for plant transformation. Compared to other promoters, the egg cell-specific (*EC1.1*) promoter was found to be more efficient for gene targeting of the *ALS* gene. In the T1 generation, 55 out of 74 lines (74%) generated heritable GT events and the majority of the lines segregated for herbicide-resistant T2 plants at a range of 1% [82]. In a different study, the DD45 promoter-driven Cas9 gene was assembled in a vector for plant transformation and expression before the delivery of donor templates and sgRNAs for precise gene targeting in subsequent generations [81]. Two endogenous DNA glycosylase genes, *Repressor Of Silencing 1* (*ROS1*) and *DEMETER* (*DME*), were targeted for green fluorescent protein (GFP) fusion or fragment replacement using this sequential transformation strategy. Successful gene targeting events were identified at a frequency of 5.8–9.1% using bulked T2 populations and positive individuals were shown to segregate at a frequency of 6.5%–88.3% from candidate T2 populations. This frequency is remarkable considering that no selection marker was used to assist the screening for GT plants.

In addition to the aforementioned methods, many interesting approaches tested in bacteria and mammalian cells have not been used in plants yet. In view of the fact that the cleavage activity of CRISPR/Cas9 systems is quite high *in vivo*, it is thought that one of the rate-limiting steps in precise gene editing can be the availability of repair templates. To increase the accessibility of donor templates to induced DSBs, a possible strategy is to tether the DNA repair templates to the CRISPR/Cas9 RNP. The assembly of such ternary complexes is usually performed *in vitro* before cell transfection to guarantee the co-localization of all required gene editing components at the targeted site. Theoretically, either guide RNAs or Cas9 proteins can be the object for tethering, but the repair effects of different complexes might be affected by their spatial configuration resulting from different linkage methods. For example, an sgRNA-fused RNA aptamer-streptavidin module, termed S1mplex, was engineered to bridge CRISPR-Cas9 RNPs with a biotinylated nucleic acid donor template. Such tailored S1mplexes increased the ratio of precisely edited to imprecisely edited alleles up to 18-fold higher than standard RNP methods; however, the total HdR percentage did not seem to be improved [83]. Other studies showed that covalent linkage of donor templates to the Cas9 protein via a SNAP tag [84] or Porcine Circovirus 2 Rep protein [85] enhanced the HdR efficiency by up to 24- and 30-fold, respectively. Moreover, in the latter case, no chemical modification of the DNA repair templates was required for protein linkage, facilitating the *in vitro* assembly of the repair complexes.

However, for plant gene editing, the application of RNPs is constrained within a small fraction of plant species, which can be regenerated from protoplasts or transformed using bombardment methods. In contrast, *in vivo* tethering of donor templates to CRISPR/Cas9 complexes might be an attractive strategy to test. Recently, Sharon *et al*. developed a highly efficient method, termed Cas9 Retron Precise Parallel Editing via homologY (CRISPEY), for high-throughput precise gene editing in yeast [86]. This study makes use of bacterial retron elements to generate donor DNAs for DSB repair. In the presence of a reverse transcriptase, multicopy single-stranded DNA products will be produced and covalently tethered to their template RNA. By fusing these donor-containing retron elements to sgRNA transcripts, the assembly of gene repair complexes can be achieved *in vivo*.

### Knock-in strategies

Although the gene targeting efficiency has been increased by one to two orders of magnitude with the assistance of CRISPR systems and new gene delivery strategies, the chance of obtaining heritable gene targeting events is still quite low for most plants. The reason for this is that the end-joining pathway seems to be more efficient than the HdR pathway for DSB repair in somatic cells [87]. Therefore, if the end-joining pathway can be harnessed for targeted knock-in, a high gene insertion or replacement frequency might be achievable in plant cells.

To test this hypothesis, Li *et al*. developed a strategy to replace the second exon of the rice endogenous gene 5-enolpyruvylshikimate-3-phosphate synthase (EPSPS) via the end-joining pathway [88]. To initiate the DSB repair process, two sgRNAs were designed to target the intron regions instead of the exons, considering that the intron regions are usually less sensitive to small sequence variations. A donor template containing the desired gene substitution as well as the sgRNA targets was provided, along with the CRISPR construct, by bombardment. Although seamless gene replacements were identified at a frequency of 2.0%, most of the editing events (∼80%) were just small indels within the two sgRNA target loci.

In animal systems, the efficiency of homology-independent targeted integration has been greatly improved by using circularized donor DNA devoid of a bacterial backbone (minicircles) [89]. About 56% of transfected neuron cells contained the anticipated GFP integration and the majority of these GFP positive cells did not show indels at the integration sites. This study highlights the possibility of using minicircle DNA for plant donor DNA delivery. Compared to large DNA fragment insertions, targeted integration of small DNA fragments can be relatively easy using blunt-end double-stranded DNA oligos with phosphorothioate-modified ends, a commonly used strategy to increase the *in vivo* stability of synthesized DNA oligos [90]. These results suggest that the stability of the donor DNA may be an important factor for efficient gene integration in plants.

### Base editing

**Figure 3.**
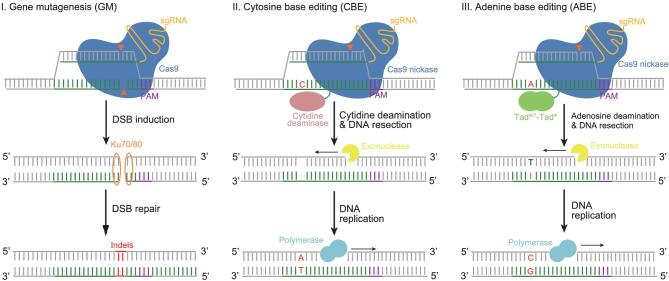
A model of CRISPR/Cas-mediated gene mutagenesis and base editing. Mechanisms of target binding, DNA cleavage and repair during gene mutagenesis (left), cytosine base editing (middle) and adenine base editing (right). Red triangles indicate the single-stranded break within the guide RNA recognition sites.

Gene targeting and knock-in require the supply of donor DNA along with the CRISPR cassette to direct the repair of DSBs. Although many strategies have been tested, efficient delivery of donor DNA as a repair template into germline cells for heritable precise gene editing remains challenging in most plants. Moreover, DSB repair outcomes are usually indels of variable sizes rather than substitutions, leading to out-of-frame mutations of the target genes [20]. Many important agronomic traits involve only one or a few base changes within the target genes [91]. Therefore, it is highly desirable to adapt the CRISPR systems for precise base substitutions (i.e. base editing) in a DSB- and template-independent manner (Fig. 3).

Base editing was first achieved by fusing a cytidine deaminase to the nickase form of Cas9 (Cas9n) [92]. This fusion protein has two biochemical activities. One is to catalyze the deamination of cytosine within a narrow window of the non-targeting strand (fifth to eighth nucleotides in the protospacer), converting cytosine to uracil. Another is to produce a single-stranded DNA break in the targeting strand to activate the G–A conversion in the opposite stand via DNA replication. The use of Cas9n increases the activity of the cytosine base editor (CBE). However, the conversion from cytosine to uracil is inhibited by endogenous uracil glycosylases, which recognize unnatural U–G pairing by catalyzing the removal of uracil. Indeed, it was shown that the editing efficiency and accuracy of the CBE system can be further improved in the presence of the uracil glycosylase inhibitor (UGI) [93].

To further expand the base-editing tool box, David Liu's laboratory developed another base editor to enable the conversion of adenine to guanine [94]. For this purpose, they used accelerated evolution methods to produce a novel protein capable of catalyzing the deamination of adenine in DNA molecules from an *Escherichia coli* adenosine deaminase, ecTadA. After deamination, the original adenine was converted to hypoxanthine, which is recognized as guanine during DNA replication. Similar to CBE, this adenine base editor (ABE) also requires a Cas9 nickase to help with target recognition and T–C conversion in the targeting stand.

Extensive studies have been performed to optimize the activity of the two base editors in a variety of plant species, including rice, wheat, maize, rapeseed, tomato, watermelon and *Arabidopsis* (Table 2). The reported editing efficiencies and accuracies of different base editing systems show a large degree of variation. As an example, three studies in rice reported that precise base substitutions were induced at a frequency of up to ∼40% using a similar rat apolipoprotein B mRNA editing enzyme, catalytic polypeptide 1 (APOBEC1)-based CBE system [95–97]. While two of the studies suggested that imprecise editing is frequently induced at the targeted loci [96,97], the other reported that indels are seldom detected [95]. Meanwhile, large variations in editing efficiency were observed when different gene loci or different plant species were targeted by the same CBE system, suggesting that the activity of CBE systems might be affected by unknown factors [95]. In addition to the rat APOBEC1 (rAPO1), the lamprey cytidine deaminase (pmCDA1) [98] and an engineered human activation-induced cytidine deaminase (hAID) [99] have also been harnessed for plant base editing by fusion to Cas9 variants. The pmCDA1-based CBE systems induced a high frequency of gene substitutions (26/45) as well as indels (21/45) at the targeted tomato genes in the T0 generation. However, its activity was apparently lower at the rice ALS locus (3.4%). In cases when the hAID was used for cytosine conversion, the editing frequencies of some tested loci were higher compared to the rAPO1-based system, but the occurrence of unexpected gene mutations was also increased. Factors affecting the editing efficiency of different CBE systems can be quite complex and require further investigation. It has been shown that the editing efficiency at the same gene locus in different plant species can also be very different when similar CBE systems are applied. For example, the rAPO1-based CBE system only induced 1.7% C–T editing at the ALS Pro197 locus in *Arabidopsis* T1 transgenic lines [100], while the editing efficiency at the same locus was 23% in watermelon [101].

**Table 2.** Summary of the efficiencies of different plant base editing tools.

							Transformation	Gene	Precise editing	Imprecise	Editing	
System type	Base editor	Guide	Promoter	Cas9	UGI	Species	method	target	rate	editing rate	window	Reference
ABE	ABE6.3	AtU6::sgRNA	35S	Cas9n		*Arabidopsis*	*Agrobacterium*	**FT**	85%	<0.1%	5–9	[102]
	ABE7.8		AtYAO					ALS				
	ABE7.9		**AtRPS5a**					LFY				
	**ABE7.10**							PDS				
	ABE7.8	OsU6::sgRNA	ZmUBI	Cas9		Rice	*Agrobacterium*	MPK6	17.64%	ND	4–6	[116]
	**ABE7.10**			**Cas9n**				MPK13	6.45%			
								SERK2	32.05%			
								**WRKY45**	62.26%			
								Tms9–1	0%			
	ABE7.10	OsU6::SpsgRNA	ZmUBI	SpCas9n		Rice	*Agrobacterium*	SPL14	4.8–61.3%	ND	5–10	[117]
		OsU6::SasgRNA		**SaCas9n**				SPL16				
								**SPL17**				
								SPL18				
								SLR1				
								LOC_Os02g247				
	ABE7.10	**OsU6::SpsgRNA**	ZmUBI	**VQR-SpCas9n**		Rice	*Agrobacterium*	SPL14	0–74.3%	ND	3–14	[17]
		OsU6::SpsgRNA		VRER-SpCas9n				SPL16				
		OsU6::SasgRNA		KKH-SaCas9n				**SPL17**				
								SPL18				
								GRF4				
								TOE1				
								IDS1				
								OMTN1				
								SNB				
								SPL13				
	ABE7.10	OsU3::sgRNA	ZmUBI	SpCas9n		Rice	*Agrobacterium*	ACC	3.2–59.1%	<0.1%	4–8	[118]
		**OsU3::esgRNA**						DEP1				
		OsU3::tRNA-sgRNA						NRT1.1B				
	ABE7.10	TaU6::sgRNA	ZmUBI	SpCas9n		Wheat	Biolistic	DEP1	0.4–1.1%			
		**TaU6-esgRNA**						GW2				
		TaU6-tRNA-sgRNA										
CBE	PmCDA1-At	OsU6::sgRNA	35S	dCas9-Os		Rice	*Agrobacterium*	**GFP**	3.41%–18.3%	NA		[98]
				**Cas9n-Os**				ALS				
	PmCDA1-At	AtU6::sgRNA	35S	Cas9n-At		Tomato	*Agrobacterium*	DELLA	Avg. 57.8%	Avg. 46.6%	1–3	
	PmCDA1-Hs							ETR1				
	rAPOBEC1	OsU3::sgRNA	ZmUBI	dCas9	UGI	Rice	*Agrobacterium*	**CDC48**	43.48%	<0.06%	3–8	[95]
				Cas9n				NRT1.1B				
								SPL14				
						Wheat	Biolistic	LOX2	1%	ND		
						Maize	*Agrobacterium*	CENH3	10%			
	rAPOBEC1	OsU6::sgRNA	ZmUBI	Cas9n		Rice	*Agrobacterium*	NRT1.1B	2.70%	10.80%	4–8	[97]
								SLR1	13.30%	8.90%		
	rAPOBEC1	OsU3::sgRNA	ZmUBI	Cas9n	UGI	Rice	*Agrobacterium*	PDS	2–40%	18%-25%	1–10	[96]
								**SBEIIb**				
	rAPOBEC1	AtU6::sgRNA	EC1	Cas9n	UGI	*Arabidopsis*	*Agrobacterium*	ALS	1.7%	NA	4–9, PAM	[100]
	rAPOBEC1	OsU6::sgRNA	35S	Cas9n	UGI	Rice	*Agrobacterium*	SERK1	17.0%	ND	2–8	[119]
			ZmUBI	VQR-Cas9n				SERK2	10.5%			
								**SPL14**	38.90%			
								pi-ta	18.20%			
	hAID	OsU6::sgRNA	ZmUBI	Cas9n		Rice	*Agrobacterium*	pi-d2kit	30.80%	19.2%	3–7	[99]
								FLS2	57%	81.90%		
	hAID	OsU6::sgRNA	ZmUBI	Cas9n	UGI			AOS1	23.30%	7.30%	1–12	
								JAR1	21.70%	5.80%		
								JAR2	11.80%	0%		
								**COI2**	69.40%	27.80%		
	rAPOBEC1	OsU6::sgRNA	ZmUBI	Cas9n	UGI			AOS1	8.30%	2.10%	3–9	
								JAR1	17.00%	2.10%		
								JAR2	13.30%	3.30%		
								COI2	73.30%	13.50%		
	rAPOBEC1	AtU6::sgRNA	35S	Cas9n	UGI	Watermelon	*Agrobacterium*	ALS	23.0%	NA	7–8	[101]
	rAPOBEC1	OsU6::SpsgRNA	ZmUBI	**SpCas9n**	UGI	Rice	*Agrobacterium*	PMS1	0–80%	ND	4–15	[17]
		OsU6::SpsgRNA		**VQR-SpCas9n**			Biolistic	**PMS3**				
		OsU6::SasgRNA		KKH-SaCas9n				SPL14				
								SPL17				
								**SNB**				

Bold letters are used when there were exceptionally good base editing effects. ND: not detected; NA: not available.

The ABE systems have also been tested in plant cells. Compared to the ABE 7.10 system, which showed robust editing activities in rice and *Arabidopsis*, the editing activity of the ABE7.8 system was almost non-existent [102]. In contrast to CBE systems, which tend to generate highly frequent but imprecise editing at the target locus, almost no indels were identified with ABE systems (Table 2). Promoter activity seems to be critical for efficient base editing in *Arabidopsis* since the use of the ribosomal protein S5A (*RPS5A*) promoter to drive the expression of ABE7.10 resulted in 85% editing efficiency at target loci in T1 plants, while the CaMV 35S and Yao promoters produced almost no mutations [102].

Although there is ample room for improvement, base editors can be very efficient and easy to use for precise gene editing. A shortcoming is that their activity is constrained to a narrow window defined by the PAM motif. This limitation can be overcome by using different Cas9 variants to extend the spectrum of gene targets [17].

## CONCLUDING REMARKS

With the emergence of new CRISPR-based tools, targeted gene editing has become increasingly efficient and flexible in plant cells. In addition to targeted gene mutagenesis, other types of genetic modifications, such as base substitution, gene knock-in and replacement, have become achievable in multiple plant species (Tables 1 and 2). New advances in gene targeting strategies now theoretically allow precise gene editing to be achieved at any locus without requiring selection markers. Together, these technical advances promise any kind of editing at targeted gene loci in model plants, thus expanding the scope of the application of CRISPR systems for genetic studies.

In general, CRISPR-based gene editing tools can be classified into two categories: gene-mutagenesis tools and gene-correction tools. The first category is usually used to introduce full or partial loss-of-function mutations into the target loci, such as the canonical CRISPR/Cas9 systems and the base editor systems. Random indels or substitutions in coding sequences or intron splicing sites can cause frameshifts or alternative splicing of target genes. In addition, many regulatory elements located in non-coding regions are also required for fine regulation of gene function. Many of these elements have been difficult to study due to the lack of a controllable mutagen, but can now be dissected readily using CRISPR. With the development of CRISPR-based gene-mutagenesis tools, forward genetics screens can now be performed at scales ranging from single genes to genome-wide.

However, for gene-correction purposes, the modifications on the targeted gene loci need to be precise. Tools in this category include the two base editing systems as well as fragment deletion, insertion and replacement tools. The later three tools are usually adapted from dual-target CRISPR systems; however, in the absence of a repair template, fragment deletions or reversions are induced at a much higher efficiency than insertion or replacement. Expression of the CRISPR elements in appropriate tissues and developmental stages is important for the efficiency of small indel mutations and base editing. However, to perform precise gene editing with the guidance of donor DNA, every step from DSB induction to donor DNA supply is critical. Although much progress has been achieved over recent years, the low HdR frequency in plant cells is still a bottleneck for precise gene editing.

With the aforementioned technical advances, the application of plant gene editing tools is being gradually broadened from genetic research to crop breeding. For crop breeding purposes, one big challenge is to achieve efficient delivery of CRISPR components into reproductive cells to generate heritable gene modifications. For transformable plants, genetic transformation methods can be quite efficient at delivering foreign genes into plant reproductive cells, but the associated tissue culture and regeneration steps are often technically demanding and time-consuming. Moreover, many crop species and elite varieties are recalcitrant or extremely difficult to transform. Tissue culture- and plant regeneration-independent technologies for delivering gene editing reagents are needed in order to apply the powerful gene editing tools to all plants. Another big challenge for crop breeding by gene editing is to decide which gene(s) to edit in order to improve a particular trait. Many important agronomic traits are polygenic in nature, and their genetic basis is difficult to dissect, partly due to complex genetic interactions. With the aid of CRISPR gene editing tools, the underlying genes for complex agronomic traits will be identified, which then can be edited for crop improvement.

When used for crop breeding, CRISPR cassettes need to be removed from the crop genome after gene editing has been achieved as a likely prerequisite to gain regulatory approval of CRISPR-edited crops for commercial applications. Transgene-free gene-edited plants can be obtained by genetic segregation during sexual reproduction. To obtain transgene-free edited plants more efficiently, a negative selection marker could be useful. Examples of such negative selection include a seed-specific fluorescence protein expression cassette [103] and an early embryo-specific toxic protein expression cassette [104]. The use of IVTs and RNPs can obviate this problem as no foreign DNA is introduced into the plant genome.

In spite of the overwhelming advantages provided by CRISPR technologies for crop improvement, the application of the technologies could be prohibited in some countries by government policies that regulate gene-edited products as GMOs. Scientifically, it is illogical to regulate systems that produce single, very precise genomic changes, while others, such as chemical and radiation mutagenesis, which produces thousands of random mutations, remain unregulated. CRISPR and other gene editing technologies have already delivered some important advances in crop breeding and we anticipate that we have only seen the tip of the iceberg, with more exciting developments yet to come.

## References

[1] Hartwell L. *Genetics: From Genes to Genomes*, 6th ed. New York: McGraw-Hill Education, 2017.

[2] Gaj T , Gersbach CA, Barbas CF III. ZFN, TALEN, and CRISPR/Cas-based methods for genome engineering. Trends Biotechnol 2013; 31: 397–405. 23664777 10.1016/j.tibtech.2013.04.004 PMC3694601

[3] Shrivastav M , De Haro LP, Nickoloff JA. Regulation of DNA double-strand break repair pathway choice. Cell Res 2008; 18: 134–47. 18157161 10.1038/cr.2007.111

[4] Ceccaldi R , Rondinelli B, D’Andrea AD. Repair pathway choices and consequences at the double-strand break. Trends Cell Biol 2016; 26: 52–64. 26437586 10.1016/j.tcb.2015.07.009 PMC4862604

[5] Jiang W , Marraffini LA. CRISPR-Cas: new tools for genetic manipulations from bacterial immunity systems. Annu Rev Microbiol 2015; 69: 209–28. 26209264 10.1146/annurev-micro-091014-104441

[6] Jinek M , Chylinski K, Fonfara I et al. A programmable dual-RNA-guided DNA endonuclease in adaptive bacterial immunity. Science 2012; 337: 816–21. 22745249 10.1126/science.1225829 PMC6286148

[7] Hsu PD , Lander ES, Zhang F. Development and applications of CRISPR-Cas9 for genome engineering. Cell 2014; 157: 1262–78. 24906146 10.1016/j.cell.2014.05.010 PMC4343198

[8] Belhaj K , Chaparro-Garcia A, Kamoun S et al. Plant genome editing made easy: targeted mutagenesis in model and crop plants using the CRISPR/Cas system. Plant Methods 2013; 9: 39.24112467

[9] Mojica FJ , Diez-Villasenor C, Garcia-Martinez J et al. Short motif sequences determine the targets of the prokaryotic CRISPR defence system. Microbiology 2009; 155: 733–40. 19246744 10.1099/mic.0.023960-0

[10] Murugan K , Babu K, Sundaresan R et al. The revolution continues: newly discovered systems expand the CRISPR-Cas toolkit. Mol Cell 2017; 68: 15–25. 28985502 10.1016/j.molcel.2017.09.007 PMC5683099

[11] Steinert J , Schiml S, Fauser F et al. Highly efficient heritable plant genome engineering using Cas9 orthologues from Streptococcus thermophilus and Staphylococcus aureus. Plant J 2015; 84: 1295–305. 26576927 10.1111/tpj.13078

[12] Zetsche B , Gootenberg JS, Abudayyeh OO et al. Cpf1 is a single RNA-guided endonuclease of a class 2 CRISPR-Cas system. Cell 2015; 163: 759–71. 26422227 10.1016/j.cell.2015.09.038 PMC4638220

[13] Zetsche B , Heidenreich M, Mohanraju P et al. Multiplex gene editing by CRISPR-Cpf1 using a single crRNA array. Nat Biotechnol 2017; 35: 31–4. 27918548 10.1038/nbt.3737 PMC5225075

[14] Mitsunobu H , Teramoto J, Nishida K et al. Beyond native Cas9: manipulating genomic information and function. Trends Biotechnol 2017; 35: 983–96. 28739220 10.1016/j.tibtech.2017.06.004

[15] Nishimasu H , Shi X, Ishiguro S et al. Engineered CRISPR-Cas9 nuclease with expanded targeting space. Science 2018; 361: 1259–62. 30166441 10.1126/science.aas9129 PMC6368452

[16] Endo M , Mikami M, Endo A et al. Genome editing in plants by engineered CRISPR–Cas9 recognizing NG PAM. Nat Plants 2019; 5: 14–7. 30531939 10.1038/s41477-018-0321-8

[17] Hua K , Tao X, Zhu JK. Expanding the base editing scope in rice by using Cas9 variants. Plant Biotechnol J 2018; 17: 499–504. 10.1111/pbi.12993 PMC6335069 30051586

[18] Kim S , Bae T, Hwang J et al. Rescue of high-specificity Cas9 variants using sgRNAs with matched 5′ nucleotides. Genome Biol 2017; 18: 218. 29141659 10.1186/s13059-017-1355-3 PMC5686910

[19] Zhang D , Zhang H, Li T et al. Perfectly matched 20-nucleotide guide RNA sequences enable robust genome editing using high-fidelity SpCas9 nucleases. Genome Biol 2017; 18: 191. 29020979 10.1186/s13059-017-1325-9 PMC5637269

[20] Feng Z , Mao Y, Xu N et al. Multigeneration analysis reveals the inheritance, specificity, and patterns of CRISPR/Cas-induced gene modifications in Arabidopsis. Proc Natl Acad Sci USA 2014; 111: 4632–7. 24550464 10.1073/pnas.1400822111 PMC3970504

[21] Lu Y , Ye X, Guo R et al. Genome-wide targeted mutagenesis in rice using the CRISPR/Cas9 system. Mol Plant 2017; 10: 1242–5. 28645638 10.1016/j.molp.2017.06.007

[22] Meng X , Yu H, Zhang Y et al. Construction of a genome-wide mutant library in rice using CRISPR/Cas9. Mol Plant 2017; 10: 1238–41. 28645639 10.1016/j.molp.2017.06.006

[23] Shalem O , Sanjana NE, Zhang F. High-throughput functional genomics using CRISPR-Cas9. Nat Rev Genet 2015; 16: 299–311. 25854182 10.1038/nrg3899 PMC4503232

[24] Zhang H , Zhang JS, Lang ZB et al. Genome editing-principles and applications for functional genomics research and crop improvement. Crit Rev Plant Sci 2017; 36: 291–309.

[25] Shu QY , Forster BP, Nakagawa H. Plant Mutation Breeding and Biotechnology. Wallingford: CABI Publishing, 2012.

[26] Fraley RT , Rogers SG, Horsch RB et al. Expression of bacterial genes in plant cells. Proc Natl Acad Sci USA 1983; 80: 4803–7. 6308651 10.1073/pnas.80.15.4803 PMC384133

[27] Saeglitz C , Bartsch D. Regulatory and associated political issues with respect to Bt transgenic maize in the European Union. J Invertebr Pathol 2003; 83: 107–9. 12788278 10.1016/s0022-2011(03)00062-4

[28] Waltz E . Gene-edited CRISPR mushroom escapes US regulation. Nature 2016; 532: 293. 27111611 10.1038/nature.2016.19754

[29] Callaway E . CRISPR plants now subject to tough GM laws in European Union. Nature 2018; 560: 16. 30065322 10.1038/d41586-018-05814-6

[30] Cyranoski D , Ledford H. Genome-edited baby claim provokes international outcry. Nature 2018; 563: 607–8. 30482929 10.1038/d41586-018-07545-0

[31] Fu Y , Foden JA, Khayter C et al. High-frequency off-target mutagenesis induced by CRISPR-Cas nucleases in human cells. Nat Biotechnol 2013; 31: 822–6. 23792628 10.1038/nbt.2623 PMC3773023

[32] Hsu PD , Scott DA, Weinstein JA et al. DNA targeting specificity of RNA-guided Cas9 nucleases. Nat Biotechnol 2013; 31: 827–32. 23873081 10.1038/nbt.2647 PMC3969858

[33] Pattanayak V , Lin S, Guilinger JP et al. High-throughput profiling of off-target DNA cleavage reveals RNA-programmed Cas9 nuclease specificity. Nat Biotechnol 2013; 31: 839–43. 23934178 10.1038/nbt.2673 PMC3782611

[34] Feng Z , Zhang B, Ding W et al. Efficient genome editing in plants using a CRISPR/Cas system. Cell Res 2013; 23: 1229–32. 23958582 10.1038/cr.2013.114 PMC3790235

[35] Zhang H , Zhang J, Wei P et al. The CRISPR/Cas9 system produces specific and homozygous targeted gene editing in rice in one generation. Plant Biotechnol J 2014; 12: 797–807. 24854982 10.1111/pbi.12200

[36] Tang X , Liu GQ, Zhou JP et al. A large-scale whole-genome sequencing analysis reveals highly specific genome editing by both Cas9 and Cpf1 (Cas12a) nucleases in rice. Genome Biol 2018; 19: 84. 29973285 10.1186/s13059-018-1458-5 PMC6031188

[37] Nekrasov V , Wang CM, Win J et al. Rapid generation of a transgene-free powdery mildew resistant tomato by genome deletion. Sci Rep 2017; 7: 482. 10.1038/s41598-017-00578-x PMC5428673 28352080

[38] Liu H , Ding Y, Zhou Y et al. CRISPR-P 2.0: an improved CRISPR-Cas9 tool for genome editing in plants. Mol Plant 2017; 10: 530–2. 28089950 10.1016/j.molp.2017.01.003

[39] Xie X , Ma X, Zhu Q et al. CRISPR-GE: a convenient software toolkit for CRISPR-based genome editing. Mol Plant 2017; 10: 1246–9. 28624544 10.1016/j.molp.2017.06.004

[40] Slaymaker IM , Gao LY, Zetsche B et al. Rationally engineered Cas9 nucleases with improved specificity. Science 2016; 351: 84–8. 26628643 10.1126/science.aad5227 PMC4714946

[41] Kleinstiver BP , Pattanayak V, Prew MS et al. High-fidelity CRISPR-Cas9 nucleases with no detectable genome-wide off-target effects. Nature 2016; 529: 490–5. 26735016 10.1038/nature16526 PMC4851738

[42] Wang M , Lu Y, Botella JR et al. Gene targeting by homology-directed repair in rice using a geminivirus-based CRISPR/Cas9 system. Mol Plant 2017; 10: 1007–10. 28315751 10.1016/j.molp.2017.03.002

[43] Liu W , Zhu X, Lei M et al. A detailed procedure for CRISPR/Cas9-mediated gene editing in Arabidopsis thaliana. Sci Bull 2015; 60: 1332–47.

[44] Mao Y , Zhang Z, Feng Z et al. Development of germ-line-specific CRISPR-Cas9 systems to improve the production of heritable gene modifications in Arabidopsis. Plant Biotechnol J 2016; 14: 519–32. 26360626 10.1111/pbi.12468 PMC5515382

[45] Wang ZP , Xing HL, Dong L et al. Egg cell-specific promoter-controlled CRISPR/Cas9 efficiently generates homozygous mutants for multiple target genes in Arabidopsis in a single generation. Genome Biol 2015; 16: 144. 26193878 10.1186/s13059-015-0715-0 PMC4507317

[46] Mao Y , Botella JR, Zhu JK. Heritability of targeted gene modifications induced by plant-optimized CRISPR systems. Cell Mol Life Sci 2017; 74: 1075–93. 27677493 10.1007/s00018-016-2380-1 PMC11107718

[47] Mao Y , Yang X, Zhou Y et al. Manipulating plant RNA-silencing pathways to improve the gene editing efficiency of CRISPR/Cas9 systems. Genome Biol 2018; 19: 149. 30266091 10.1186/s13059-018-1529-7 PMC6161460

[48] Ran YD , Liang Z, Gao CX. Current and future editing reagent delivery systems for plant genome editing. Sci China Life Sci 2017; 60: 490–505. 28527114 10.1007/s11427-017-9022-1

[49] Mikami M , Toki S, Endo M. Parameters affecting frequency of CRISPR/Cas9 mediated targeted mutagenesis in rice. Plant Cell Rep 34: 1807–15. 10.1007/s00299-015-1826-5 26134856

[50] Ma X , Zhang Q, Zhu Q et al. A robust CRISPR/Cas9 system for convenient, high-efficiency multiplex genome editing in monocot and dicot plants. Mol Plant 2015; 8: 1274–84. 25917172 10.1016/j.molp.2015.04.007

[51] Chen L , Li W, Katin-Grazzini L et al. A method for the production and expedient screening of CRISPR/Cas9-mediated non-transgenic mutant plants. Hortic Res 2018; 5: 13. 29531752 10.1038/s41438-018-0023-4 PMC5834642

[52] Liang Z , Chen K, Zhang Y et al. Genome editing of bread wheat using biolistic delivery of CRISPR/Cas9 in vitro transcripts or ribonucleoproteins. Nat Protoc 2018; 13: 413–30. 29388938 10.1038/nprot.2017.145

[53] Woo JW , Kim J, Kwon SI et al. DNA-free genome editing in plants with preassembled CRISPR-Cas9 ribonucleoproteins. Nat Biotechnol 2015; 33: 1162–4. 26479191 10.1038/nbt.3389

[54] Svitashev S , Schwartz C, Lenderts B et al. Genome editing in maize directed by CRISPR-Cas9 ribonucleoprotein complexes. Nat Commun 2016; 7: 13274. 27848933 10.1038/ncomms13274 PMC5116081

[55] Liang Z , Chen K, Li T et al. Efficient DNA-free genome editing of bread wheat using CRISPR/Cas9 ribonucleoprotein complexes. Nat Commun 2017; 8: 14261. 28098143 10.1038/ncomms14261 PMC5253684

[56] Liu Q , Chen B, Wang Q et al. Carbon nanotubes as molecular transporters for walled plant cells. Nano Lett 2009; 9: 1007–10. 19191500 10.1021/nl803083u

[57] Hussain HI , Yi Z, Rookes JE et al. Mesoporous silica nanoparticles as a biomolecule delivery vehicle in plants. J Nanopart Res 2013; 15: 1676.

[58] Kurepa J , Paunesku T, Vogt S et al. Uptake and distribution of ultrasmall anatase TiO_2_ Alizarin red S nanoconjugates in *Arabidopsis thaliana*. Nano Lett 2010; 10: 2296–302. 20218662 10.1021/nl903518f PMC2912449

[59] Cunningham FJ , Goh NS, Demirer GS et al. Nanoparticle-mediated delivery towards advancing plant genetic engineering. Trends Biotechnol 2018; 36: 882–97. 29703583 10.1016/j.tibtech.2018.03.009 PMC10461776

[60] Liu J , Wang F-H, Wang L-L et al. Preparation of fluorescence starch-nanoparticle and its application as plant transgenic vehicle. J Cent South Univ Technol 2008; 15: 768–73.

[61] Zhao X , Meng Z, Wang Y et al. Pollen magnetofection for genetic modification with magnetic nanoparticles as gene carriers. Nat Plants 2017; 3: 956–64. 29180813 10.1038/s41477-017-0063-z

[62] Lloyd J , Meinke D. A comprehensive dataset of genes with a loss-of-function mutant phenotype in Arabidopsis. Plant Physiol 2012; 158: 1115–29. 22247268 10.1104/pp.111.192393 PMC3291275

[63] Wang H , Yang H, Shivalila CS et al. One-step generation of mice carrying mutations in multiple genes by CRISPR/Cas-mediated genome engineering. Cell 2013; 153: 910–8. 23643243 10.1016/j.cell.2013.04.025 PMC3969854

[64] Ma X , Zhu Q, Chen Y et al. CRISPR/Cas9 platforms for genome editing in plants: developments and applications. Mol Plant 2016; 9: 961–74. 27108381 10.1016/j.molp.2016.04.009

[65] Xie K , Minkenberg B, Yang Y. Boosting CRISPR/Cas9 multiplex editing capability with the endogenous tRNA-processing system. Proc Natl Acad Sci USA 2015; 112: 3570–5. 25733849 10.1073/pnas.1420294112 PMC4371917

[66] Cermak T , Curtin SJ, Gil-Humanes J et al. A multipurpose toolkit to enable advanced genome engineering in plants. Plant Cell 2017; 29: 1196–217. 28522548 10.1105/tpc.16.00922 PMC5502448

[67] Zhang Z , Mao Y, Ha S et al. A multiplex CRISPR/Cas9 platform for fast and efficient editing of multiple genes in Arabidopsis. Plant Cell Rep 2016; 35: 1519–33. 26661595 10.1007/s00299-015-1900-z PMC5512712

[68] Ding D , Chen K, Chen Y et al. Engineering introns to express RNA guides for Cas9- and Cpf1-mediated multiplex genome editing. Mol Plant 2018; 11: 542–52. 29462720 10.1016/j.molp.2018.02.005

[69] Tang X , Zheng X, Qi Y et al. A single transcript CRISPR-Cas9 system for efficient genome editing in plants. Mol Plant 2016; 9: 1088–91. 27212389 10.1016/j.molp.2016.05.001

[70] Mikami M , Toki S, Endo M. In planta processing of the SpCas9-gRNA complex. Plant Cell Physiol 2017; 58: 1857–67. 29040704 10.1093/pcp/pcx154 PMC5921533

[71] Wang M , Mao Y, Lu Y et al. Multiplex gene editing in rice with simplified CRISPR-Cpf1 and CRISPR-Cas9 systems. J Integr Plant Biol 2018; 60: 626–31. 29762900 10.1111/jipb.12667

[72] Britt AB , May GD. Re-engineering plant gene targeting. Trends Plant Sci 2003; 8: 90–5. 12597876 10.1016/S1360-1385(03)00002-5

[73] Puchta H , Dujon B, Hohn B. Homologous recombination in plant cells is enhanced by in vivo induction of double strand breaks into DNA by a site-specific endonuclease. Nucl Acids Res 1993; 21: 5034–40. 8255757 10.1093/nar/21.22.5034 PMC310614

[74] Steinert J , Schiml S, Puchta H. Homology-based double-strand break-induced genome engineering in plants. Plant Cell Rep 2016; 35: 1429–38. 27084537 10.1007/s00299-016-1981-3

[75] Schiml S , Fauser F, Puchta H. The CRISPR/Cas system can be used as nuclease for in planta gene targeting and as paired nickases for directed mutagenesis in Arabidopsis resulting in heritable progeny. Plant J 2014; 80: 1139–50. 25327456 10.1111/tpj.12704

[76] Svitashev S , Young JK, Schwartz C et al. Targeted mutagenesis, precise gene editing, and site-specific gene insertion in maize using Cas9 and guide RNA. Plant Physiol 2015; 169: 931–45. 26269544 10.1104/pp.15.00793 PMC4587463

[77] Sun Y , Zhang X, Wu C et al. Engineering herbicide-resistant rice plants through CRISPR/Cas9-mediated homologous recombination of acetolactate synthase. Mol Plant 2016; 9: 628–31. 26768120 10.1016/j.molp.2016.01.001

[78] Li J , Zhang X, Sun Y et al. Efficient allelic replacement in rice by gene editing: a case study of the NRT1.1B gene. J Integr Plant Biol 2018; 60: 536–40. 29575650 10.1111/jipb.12650

[79] Baltes NJ , Gil-Humanes J, Cermak T et al. DNA replicons for plant genome engineering. Plant Cell 2014; 26: 151–63. 24443519 10.1105/tpc.113.119792 PMC3963565

[80] Zaidi SS , Mansoor S. Viral vectors for plant genome engineering. Front Plant Sci 2017; 8: 539. 28443125 10.3389/fpls.2017.00539 PMC5386974

[81] Miki D , Zhang W, Zeng W et al. CRISPR/Cas9-mediated gene targeting in Arabidopsis using sequential transformation. Nat Commun 2018; 9: 1967. 29773790 10.1038/s41467-018-04416-0 PMC5958078

[82] Wolter F , Klemm J, Puchta H. Efficient in planta gene targeting in Arabidopsis using egg cell-specific expression of the Cas9 nuclease of Staphylococcus aureus. Plant J 2018; 94: 735–46. 29573495 10.1111/tpj.13893

[83] Carlson-Stevermer J , Abdeen AA, Kohlenberg L et al. Assembly of CRISPR ribonucleoproteins with biotinylated oligonucleotides via an RNA aptamer for precise gene editing. Nat Commun 2017; 8: 1711. 29167458 10.1038/s41467-017-01875-9 PMC5700129

[84] Savic N , Ringnalda FC, Lindsay H et al. Covalent linkage of the DNA repair template to the CRISPR-Cas9 nuclease enhances homology-directed repair. Elife 2018; 7: e33761. 10.7554/eLife.33761 PMC6023611 29809142

[85] Aird EJ , Lovendahl KN, St Martin A et al. Increasing Cas9-mediated homology-directed repair efficiency through covalent tethering of DNA repair template. Commun Biol 2018; 1: 54. 30271937 10.1038/s42003-018-0054-2 PMC6123678

[86] Sharon E , Chen SA, Khosla NM et al. Functional genetic variants revealed by massively parallel precise genome editing. Cell 2018; 175: 544–57.e16. 30245013 10.1016/j.cell.2018.08.057 PMC6563827

[87] Boyko A , Zemp F, Filkowski J et al. Double-strand break repair in plants is developmentally regulated. Plant Physiol 2006; 141: 488–97. 16474027 10.1104/pp.105.074658 PMC1475443

[88] Li J , Meng X, Zong Y et al. Gene replacements and insertions in rice by intron targeting using CRISPR-Cas9. Nat Plants 2016; 2: 16139. 27618611 10.1038/nplants.2016.139

[89] Suzuki K , Tsunekawa Y, Hernandez-Benitez R et al. In vivo genome editing via CRISPR/Cas9 mediated homology-independent targeted integration. Nature 2016; 540: 144–9. 27851729 10.1038/nature20565 PMC5331785

[90] Tsai SQ , Zheng Z, Nguyen NT et al. GUIDE-seq enables genome-wide profiling of off-target cleavage by CRISPR-Cas nucleases. Nat Biotechnol 2015; 33: 187–97. 25513782 10.1038/nbt.3117 PMC4320685

[91] Doebley JF , Gaut BS, Smith BD. The molecular genetics of crop domestication. Cell 2006; 127: 1309–21. 17190597 10.1016/j.cell.2006.12.006

[92] Komor AC , Kim YB, Packer MS et al. Programmable editing of a target base in genomic DNA without double-stranded DNA cleavage. Nature 2016; 533: 420–4. 27096365 10.1038/nature17946 PMC4873371

[93] Wang L , Xue W, Yan L et al. Enhanced base editing by co-expression of free uracil DNA glycosylase inhibitor. Cell Res 2017; 27: 1289–92. 28849781 10.1038/cr.2017.111 PMC5630677

[94] Gaudelli NM , Komor AC, Rees HA et al. Programmable base editing of A . T to G . C in genomic DNA without DNA cleavage. Nature 2017; 551: 464–71. 29160308 10.1038/nature24644 PMC5726555

[95] Zong Y , Wang Y, Li C et al. Precise base editing in rice, wheat and maize with a Cas9-cytidine deaminase fusion. Nat Biotechnol 2017; 35: 438–40. 28244994 10.1038/nbt.3811

[96] Li J , Sun Y, Du J et al. Generation of targeted point mutations in rice by a modified CRISPR/Cas9 system. Mol Plant 2017; 10: 526–9. 27940306 10.1016/j.molp.2016.12.001

[97] Lu Y , Zhu JK. Precise editing of a target base in the rice genome using a modified CRISPR/Cas9 system. Mol Plant 2017; 10: 523–5. 27932049 10.1016/j.molp.2016.11.013

[98] Shimatani Z , Kashojiya S, Takayama M et al. Targeted base editing in rice and tomato using a CRISPR-Cas9 cytidine deaminase fusion. Nat Biotechnol 2017; 35: 441–3. 28346401 10.1038/nbt.3833

[99] Ren B , Yan F, Kuang Y et al. Improved base editor for efficiently inducing genetic variations in rice with CRISPR/Cas9-guided hyperactive hAID mutant. Mol Plant 2018; 11: 623–6. 29382569 10.1016/j.molp.2018.01.005

[100] Chen Y , Wang Z, Ni H et al. CRISPR/Cas9-mediated base-editing system efficiently generates gain-of-function mutations in Arabidopsis. Sci China Life Sci 2017; 60: 520–3. 28303459 10.1007/s11427-017-9021-5

[101] Tian S , Jiang L, Cui X et al. Engineering herbicide-resistant watermelon variety through CRISPR/Cas9-mediated base-editing. Plant Cell Rep 2018; 37: 1353–6. 29797048 10.1007/s00299-018-2299-0

[102] Kang BC , Yun JY, Kim ST et al. Precision genome engineering through adenine base editing in plants. Nat Plants 2018; 4: 427–31. 29867128 10.1038/s41477-018-0178-x

[103] He Y , Wang R, Dai X et al. On improving CRISPR for editing plant genes: ribozyme-mediated guide RNA production and fluorescence-based technology for isolating transgene-free mutants generated by CRISPR. Prog Mol Biol Transl Sci 2017; 149: 151–66. 28712495 10.1016/bs.pmbts.2017.03.012

[104] He Y , Zhu M, Wang L et al. Programmed self-elimination of the CRISPR/Cas9 construct greatly accelerates the isolation of edited and transgene-free rice plants. Mol Plant 2018; 11: 1210–3. 29857174 10.1016/j.molp.2018.05.005

[105] Hahn F , Eisenhut M, Mantegazza O et al. Homology-directed repair of a defective glabrous gene in Arabidopsis with Cas9-based gene targeting. Front Plant Sci 2018; 9: 424.29675030

[106] Cermak T , Baltes NJ, Cegan R et al. High-frequency, precise modification of the tomato genome. Genome Biol 2015; 16: 232. 26541286 10.1186/s13059-015-0796-9 PMC4635538

[107] Dahan-Meir T , Filler-Hayut S, Melamed-Bessudo C et al. Efficient in planta gene targeting in tomato using geminiviral replicons and the CRISPR/Cas9 system. Plant J 2018; 95: 5–16. 29668111 10.1111/tpj.13932

[108] Li Z , Liu ZB, Xing A et al. Cas9-guide RNA directed genome editing in soybean. Plant Physiol 2015; 169: 960–70. 26294043 10.1104/pp.15.00783 PMC4587461

[109] Butler NM , Baltes NJ, Voytas DF et al. Geminivirus-mediated genome editing in potato (Solanum tuberosum L.) using sequence-specific nucleases. Front Plant Sci 2016; 7: 1045. 27493650 10.3389/fpls.2016.01045 PMC4955380

[110] Endo M , Mikami M, Toki S. Biallelic gene targeting in rice. Plant Physiol 2016; 170: 667–77. 26668334 10.1104/pp.15.01663 PMC4734577

[111] Butt H , Eid A, Ali Z et al. Efficient CRISPR/Cas9-mediated genome editing using a chimeric single-guide RNA molecule. Front Plant Sci 2017; 8: 1441. 28883826 10.3389/fpls.2017.01441 PMC5573723

[112] Begemann MB , Gray BN, January E et al. Precise insertion and guided editing of higher plant genomes using Cpf1 CRISPR nucleases. Sci Rep 2017; 7: 11606. 28912524 10.1038/s41598-017-11760-6 PMC5599503

[113] Li S , Li J, Zhang J et al. Synthesis-dependent repair of Cpf1-induced double-strand DNA breaks enables targeted gene replacement in rice. J Exp Bot 2018; 69; 4715–21. 10.1093/jxb/ery245 PMC6137971 29955893

[114] Shi J , Gao H, Wang H et al. ARGOS8 variants generated by CRISPR-Cas9 improve maize grain yield under field drought stress conditions. Plant Biotechnol J 2017; 15: 207–16. 27442592 10.1111/pbi.12603 PMC5258859

[115] Gil-Humanes J , Wang Y, Liang Z et al. High-efficiency gene targeting in hexaploid wheat using DNA replicons and CRISPR/Cas9. Plant J 2017; 89: 1251–62. 27943461 10.1111/tpj.13446 PMC8439346

[116] Yan F , Kuang Y, Ren B et al. Highly efficient A·T to G·C base editing by Cas9n-guided tRNA adenosine deaminase in rice. Mol Plant 2018; 11: 631–4. 29476918 10.1016/j.molp.2018.02.008

[117] Hua K , Tao X, Yuan F et al. Precise A·T to G·C base editing in the rice genome. Mol Plant 2018; 11: 627–30. 29476916 10.1016/j.molp.2018.02.007

[118] Li C , Zong Y, Wang Y et al. Expanded base editing in rice and wheat using a Cas9-adenosine deaminase fusion. Genome Biol 2018; 19: 59. 29807545 10.1186/s13059-018-1443-z PMC5972399

[119] Ren B , Yan F, Kuang Y et al. A CRISPR/Cas9 toolkit for efficient targeted base editing to induce genetic variations in rice. Sci China Life Sci 2017; 60: 516–9. 28260228 10.1007/s11427-016-0406-x

